# The complex interaction between oestrogen receptor genes, oestradiol, and perinatal mood

**DOI:** 10.1080/19585969.2025.2482126

**Published:** 2025-03-21

**Authors:** Gianna Zorzini, Alexandra Johann, Jelena Dukic, Ulrike Ehlert

**Affiliations:** Department of Clinical Psychology and Psychotherapy, University of Zurich, Zurich, Switzerland

**Keywords:** Oestrogen receptor, genetics, oestradiol, perinatal mood

## Abstract

**Introduction:**

Genetic variations in oestrogen receptor (ER) genes are associated with inter-individual differences in the sensitivity of ER-α, ER-β and G protein-coupled oestrogen receptor (GPER). These sensitivity differences may modulate susceptibility to mood changes during phases of endogenous oestrogen fluctuations, thereby explaining individual vulnerability. This study examined the association between ER gene variations, oestradiol and perinatal mood disturbances.

**Methods:**

A total of 159 women were observed during the perinatal period, providing saliva samples for oestradiol assessment and completing self-report measures of depressive and anxiety symptoms at five time points. Polymorphisms in ER genes were determined from dried blood spots. The associations were analysed using linear mixed models.

**Results:**

The ER-α gene haplotypes were associated with perinatal mood disturbances. The CG haplotype was associated with perinatal depressive (*p* = 0.0162, F-test) and anxiety symptoms (*p* = 2.396e-05, F-test), whereas the TA haplotype was associated with perinatal anxiety symptoms (*p* = 0.004, F-test). The interaction between ER gene variations, oestradiol and perinatal mood disturbances was not significant.

**Conclusions:**

ER-α gene variations are associated with an increased susceptibility to perinatal mood disturbances. Sensitivity differences in ER-α appear to play a more important role for emotional processes than those in ER-β and GPER, independently of oestradiol levels. This might be explained by ER-α’s more dominant expression in the hypothalamus and amygdala.

## Introduction

During the peripartum period, up to 70–80% of women experience mood disturbances, encompassing depressive and/or anxiety symptoms (Andela and Dewani 2023), which can have a profound negative impact not only on the mother but also on the child. A considerable proportion of these women meet the diagnostic criteria for a mood disorder, with prevalence estimates reaching as high as 17% for perinatal depression (Wang et al. 2021) and 15.9% for any anxiety disorder (Dennis et al. 2017). Moreover, these disturbances are linked to a wide range of adverse implications for the mother, including increased social isolation (Letourneau et al. 2017) and mortality due to suicide (Orsolini et al. 2016). Associated negative outcomes have also been observed in children, such as a lower likelihood of being breastfed (Grigoriadis et al. 2018) and potential impairments in cognitive development (Slomian et al. 2019).

So far, various psychosocial and biological factors have been hypothesised to contribute to the vulnerability to perinatal mood changes (Furtado et al. 2018; Zhao and Zhang 2020; Yu et al. 2021). Among these, oestrogen has been of particular interest for at least two reasons: First, pronounced oestrogen fluctuations are observed during the perinatal period (Kuijper et al. 2013; Vannuccini et al. 2016). Second, oestrogens seem to be involved in cognitive and emotional dysfunctions in mental disorders (Hwang et al. 2020). While there is no clear evidence of deviating oestrogen levels in women with perinatal mood disturbances (Bloch et al. 2003; Schiller et al. 2015; Yim et al. 2015), some evidence of an involvement of oestrogen sensitivity has emerged. For instance, in a study investigating affective symptoms in multiparous women following a pharmacological simulation of the perinatal hormonal state, Schiller et al. (2022) found that affective symptoms increased in women with a history of postpartum depression (PPD) but not in those without. Furthermore, most of the affected women were identified as hormone-sensitive, defined as a minimum 30% increase in affective symptoms during the pharmacological simulation. Accordingly, oestrogen sensitivity is proposed as one biological factor mediating the vulnerability to perinatal mood disturbances (Soares and Zitek 2008; Payne et al. 2009; Mehta et al. 2014; Mehta et al. 2019). This sensitivity depends on the efficacy of the oestrogen receptors’ (ERs) response to fluctuating oestrogen, which can be regulated by several influencing factors (Champagne and Curley 2008; Hua et al. 2018).

Genetic variations in the ER genes *ESR1*, *ESR2* and *GPER*, which encode for ER-α, ER-β and GPER, respectively, can alter the receptors’ structure and abundance, ultimately moderating their sensitivity to oestrogen (Figtree et al. 2009). Binding of oestrogen to the ERs activates various transcriptional processes and signalling mechanisms, which in turn regulate gene expression (Fuentes and Silveyra 2019). Given the widespread expression of ERs in the brain, particularly in the hypothalamus, hippocampus and amygdala (Osterlund and Hurd 2001; Hazell et al. 2009), the neuronal effects of oestrogen encompass interactions with neurotransmitter systems and modifications to the brain’s structure, which are thus associated with behavioural expressions such as mood (Barth et al. 2015; Prossnitz and Hathaway 2015; Del Río et al. 2018; Krolick et al. 2018).

**Figure 1.**
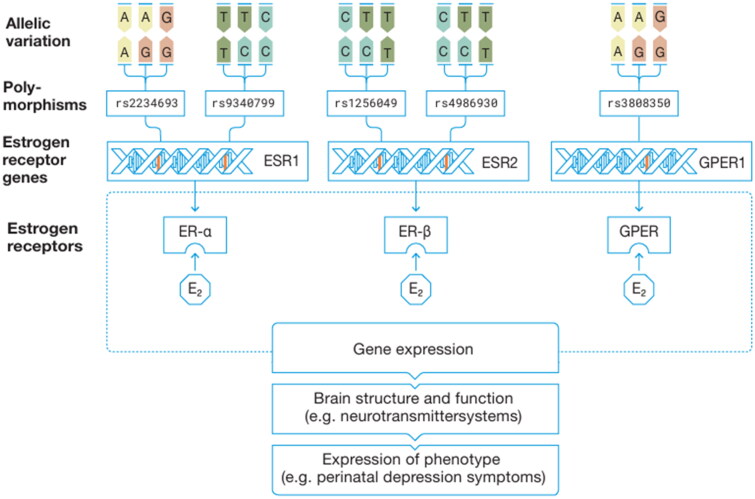
Overview of the role of oestrogen receptor gene variations in the expression of perinatal mood symptoms. E_2_: oestradiol.

Although several ER gene variations have been linked to perinatal phenotypic expressions, including depression and anxiety (Costas et al. 2010; El-Ibiary et al. 2013; Pinsonneault et al. 2013), significant research gaps remain. To date, research on *ESR1* and *ESR2* in relation to perinatal mood is limited and inconclusive, and research on *GPER* is completely lacking. Most studies have focused on individual genetic variants rather than haplotypes, a combination of frequently co-occurring alleles on a chromosome. Certain alleles are more likely to occur together than would be expected by chance, which is known as linkage disequilibrium. Therefore, investigating haplotypes may yield more reliable and robust results by providing information about additional genetic variations (Clark 2004; Slatkin 2008; Sundermann et al. 2010). Furthermore, most of the studies conducted a case-control design, with perinatal depression treated as a binary variable, thus impeding the observation of subtle mood changes and interactions over time. Although ER gene variations are hypothesised to induce inter-individual alterations in the sensitivity of the ERs, potentially modulating different responses to fluctuating oestrogen (see Figure 1), no study has yet examined whether these variations interact with oestradiol levels, ultimately resulting in different mood expressions.

The present study addressed the research gap regarding the complex interaction between ER gene variations, oestradiol and perinatal mood. Specifically, to develop a more individualised and comprehensive understanding, we aimed to identify dynamic changes and interactions implicated in the regulation of perinatal depressive and anxiety symptoms. To achieve this objective, we employed a longitudinal design, with assessments conducted at five time points during the perinatal period.

## Methods

This study was conducted as a part of a larger research project funded by the Swiss National Science Foundation and carried out at the University of Zurich, Department of Clinical Psychology and Psychotherapy. The project was approved by the Ethics Committee of the Canton of Zurich (KEK-ZH-Nr. 2018-02357) and conducted according to the principles of the Declaration of Helsinki. The project’s central aim was to investigate (epi-)genetic, biological and psychological factors implicated in female mood disorders during the transition from pregnancy to postpartum. The present study investigated the interaction between ER gene variations, oestradiol and perinatal mood, using a longitudinal design.

### Participants and procedure

Physically healthy women were recruited at 34–36 weeks of gestation and followed up until 8–12 weeks postpartum. All participants provided informed consent prior to assessment. Detailed information on the participants and the procedure can be found in previously published manuscripts (Johann and Ehlert 2020; Johann et al. 2023; Dukic et al. 2024). The original sample size (*N* = 161) was reduced to *n* = 159, as one participant withheld consent for the use of (epi-)genetic data and one participant did not provide blood samples. For the interaction analyses, the sample size was further reduced to *n* = 126, as 33 participants provided insufficient or no saliva samples (see Figure S1).

### Assessment of polymorphisms

Blood samples were collected using the dried blood spot (DBS) method, and genomic DNA was extracted using the MicroGEM forensicGEM Kit (MicroGEM UK, Southampton, United Kingdom). Five single nucleotide polymorphisms (SNPs) in three ER genes were selected for investigation: rs2234693 and rs9340799 in *ESR1*, rs1256049 and rs4986938 in *ESR2*, and rs3808350 in *GPER*. The SNPs were analysed using TaqMan SNP Genotyping Assays (ThermoFischer, Waltham, MA, USA). Genotyping was successful for all five SNPs in 158 samples. One sample failed to achieve sufficient fluorescent signal to discriminate between alleles of the SNP rs3808350 in GPER.

### Assessment of oestradiol

Over two consecutive days, participants provided four saliva samples, three in the morning (immediately after awakening, 30 and 45 min later) and one in the evening (8pm–10pm), at the following time points: 34–36 (T1) and 40 (T2) weeks gestation as well as 4–8 (T4) and 8–12 weeks postpartum (T5). Additionally, five consecutive daily samples were collected starting within 48 h after delivery (T3). Salivary oestradiol (E2; pg/mL) was quantified by luminescence immunoassay with enzyme-linked immunosorbent assay (ELISA) kits (IBL International GmbH, Hamburg, Germany, catalogue number RE62141/RE62149). E2 values were only available for *n* = 126 participants; of these, 2,154 E2 samples (32.8%) were considered to be missing (1,048 samples lay above the sensitivity threshold (>64 pg/mL) and 1,106 were completely missing). Missing E2 values were imputed using predictive mean matching in the mice package in R (version 4.3.2; R Core Team) and log-transformed.

### Assessment of perinatal mood

Depressive symptoms were assessed using the validated German version of the Edinburgh Postnatal Depression Scale (EPDS), a 10-item self-report tool that has shown good internal consistency (Bergant et al. 1998). Anxiety was assessed using the validated German-language short form of the state subscale of the State-Trait Anxiety Inventory (STAI-SKD), a five-item self-report tool that has likewise shown good internal consistency (Englert et al. 2011). Both questionnaires were completed at all assessment time points (T1–5).

### Statistical analysis

Chi-square (χ^2^) tests were used to assess the Hardy-Weinberg equilibrium (HWE). Genotypes were analysed as haplotypes, which were reconstructed by grouping SNPs by gene using an expectation-maximization algorithm (Schaid et al. 2002). Small haplotype groups, with fewer than five carriers of two copies, were combined with those of one copy. Extremely rare haplotypes, with an overall frequency of fewer than 10 occurrences, were excluded. SNPs that could not be grouped by gene were analysed separately.

For each genetic variable, a linear mixed model (LMM) was set up with continuous EPDS or STAI-SKD scores as the target variable, genetic variable (haplotype or SNP), time (T1-5), and their interactions as fixed effects, and ‘participant’ as a random effect. The haplotypes and SNPs were coded as categorical variables with levels 0, 1 and 2, according to the number of copies per participant and number of minor alleles, respectively. Time was treated as a categorical variable with five different time points (T1–5). These genetic models were each compared with a basic LMM with ‘participant’ as a random effect and time as the only fixed effect, using the likelihood ratio test (LRT). The *p*-values of the LRTs were adjusted for multiple testing using the Bonferroni-Holm method, assuming 28 tests, as a combination of seven genetic variables (two genes with three haplotypes each and one independent SNP) that were tested four times (for EPDS, STAI-SKD and for the interaction with oestradiol for both EPDS and STAI-SKD; *p* < 0.00179 = 0.05/28). In the case of significant LRT results, the model was further analysed using F-tests for fixed effects. For significant fixed effects of genetic variables and/or interactions, corresponding post-hoc pairwise comparisons were conducted.

The interaction between each genetic variable, E2 levels and perinatal depressive and anxiety symptoms were analysed similarly, by employing E2 levels and their interactions as additional fixed effects. To account for the within-day variability of E2, we used the mean values for the first day of each assessment time point. The interaction models were each compared to the corresponding genetic model using the LRT.

The model requirements were tested graphically. All tests were two-sided, with statistical significance set at *p* < 0.05.

## Results

### Sample characteristics

Sample characteristics are presented in Table S1. The sample mainly consisted of Swiss (71.7%), well-educated women (69.2% university degree) who were pregnant with their first child (55.4%). The mood measures and E2 levels at each assessed time point are displayed in Table S2. The highest mean EPDS score occurred within the first 48 h after delivery, whereas the highest mean STAI-SKD score was observed at 40 weeks of gestation.

### Genotype and haplotype distribution

The genotype distribution of all SNPs was consistent with the HWE (see Table S3). The distribution and frequencies of the reconstructed haplotypes are shown in Table S4. The two SNPs rs2234693 and rs9340799 in *ESR1* were reconstructed into three haplotypes: CG, TA and CA. Notably, the most frequent haplotype was TA, with 26.4% of participants carrying two copies and 51.6% of participants carrying one copy. The small haplotype group CA, with two copies (*n* = 3), was combined with the group with one copy. The two SNPs rs1256049 and rs4986938 in *ESR2* were reconstructed into four haplotypes: CC, CT, TC and TT. The most common haplotype was CC, with 37.7% of participants carrying two copies and 46.6% of participants carrying one copy. The extremely rare haplotype TT, with only one occurrence, was excluded from further analyses. Finally, the extremely small haplotype group TC, with two copies (*n* = 3), was combined with the group with one copy.

### Perinatal depressive symptoms and oestrogen receptor genes

**Figure 2.**
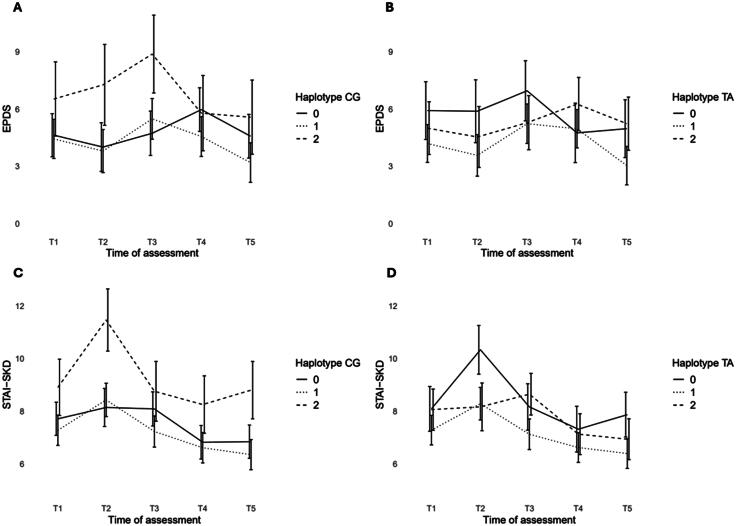
Unadjusted Edinburgh Postnatal Depression Scale (EPDS) and short form of the state subscale of the State-Trait Anxiety Inventory (STAI-SKD) scores relative to the frequencies of haplotype CG and TA in *ESR1* at five different time points across the perinatal period. Bars indicate 95% confidence intervals. For better differentiation the bars were slightly shifted. (a) Haplotype CG in *ESR1* for EPDS scores, (b) Haplotype TA in *ESR1* for EPDS scores, (c) Haplotype CG in *ESR1* for STAI-SKD scores, (d) Haplotype TA in *ESR1* for STAI-SKD scores.

Regarding the EPDS, the models with the haplotypes CG and TA in *ESR1* showed significant LRT results (see Table S5), also after including covariates (see Table S6). Figure 2a-b illustrates the depressive symptom trajectories by CG and TA haplotype frequencies across the perinatal period. After correction for multiple testing, only the model with the haplotype CG remained significant, which was further analysed using F-tests for fixed effects.

The LMM of haplotype CG showed a significant effect of haplotype (F(2, 155) = 4.13, *p* = 0.018), time (F(4, 557) = 5.02, *p* = 0.0005), and their interaction (F(8, 557) = 2.54, *p* = 0.009). Post-hoc pairwise comparisons revealed significant differences in EPDS scores between carriers of different numbers of haplotype CG at T2 and T3. Specifically, two-copy carriers showed higher EPDS scores than one-copy carriers (T2: β = –3.46, 95% CI [–6.39, –0.53], *p* = 0.014, T3: β = –3.39, 95% CI [–6.19, –0.58], *p* = 0.012) and non-carriers (T2: β = –3.26, 95% CI [–6.27, –0.24], *p* = 0.029, T3: β = –4.14, 95% CI [–7.01, –1.28], *p* = 0.002). Significant differences between the five time points were also found within each haplotype group: EPDS scores increased from T2 to T3 in one-copy carriers (β = –1.68, 95% CI [–3.34, –0.03], *p* = 0.043) and from T2 to T4 in non-carriers (β = –1.96, 95% CI [–3.79, –0.13], *p* = 0.027), and decreased from T3 to T4 in two-copy carriers (β = 3.09, 95% CI [0.14, 6.03]*, p* = 0.033) and from T3 to T5 in one-copy carriers (β = 2.29, 95% CI [0.75, 3.82]*, p* = 0.003) and two-copy carriers (β = 3.31, 95% CI [0.38, 6.22], *p* = 0.015). Additionally, follow-up logistic regression analyses were conducted to explore the association between haplotype CG and perinatal depression at each of the five assessment time points. Perinatal depression was classified using the EPDS cut-off score of ≥11 (see Table S9), which has been found to maximise both sensitivity and specificity (Levis et al. 2020). However, no significant associations were observed (all *p*-values >0.05, see Table S10).

### Perinatal anxiety and oestrogen receptor genes

Regarding the STAI-SKD, the models with the haplotype CG and TA in *ESR1* showed significant LRT results (see Table S5), also after including covariates (see Table S6). Figure 2c-d illustrates the anxiety symptom trajectories by CG and TA haplotype frequencies across the perinatal period. Both haplotype models remained significant after correction for multiple testing and were therefore further analysed using F-tests for fixed effects.

The LMM with haplotype CG showed a significant effect of haplotype (F(2, 154) = 10.64, *p* < 0.001) and time (F(4, 550) = 16.94, *p* < 0.001), but not of their interaction (F(8, 550) = 1.83, *p* = 0.069). Post-hoc pairwise comparisons revealed significant differences in STAI-SKD scores between carriers of different numbers of the haplotype CG over all time points. In detail, two-copy carriers showed higher STAI-SKD scores than one-copy carriers (β = –2.05, 95% CI [–3.14, –0.97], *p* < 0.0001) and non-carriers (β = –1.71, 95% CI [–2.82, –0.61], *p* = 0.0008).

The LMM of the haplotype TA showed a significant effect of haplotype (F(2, 154) = 5.47, *p* = 0.005), time (F(4, 549) = 17.59, *p* < 0.001), and their interaction (F(8, 549) = 2.35, *p* = 0.015). Post-hoc pairwise comparisons revealed significant differences in STAI-SKD scores between carriers of different numbers of the haplotype TA at T2, T3 and T5. Non-carriers showed higher STAI-SKD scores than one-copy carriers (T2: β = 2.05, 95% CI [0.67, 3.40]*, p* = 0.001, T5: β = 1.47, 95% CI [0.23, 2.72], *p* = 0.013) and two-copy carriers (T2: β = 2.16, 95% CI [0.58, 3.75], *p* = 0.003). At T3, two-copy carriers showed higher STAI-SKD scores than one-copy carriers (β = –1.52, 95% CI [–2.72, –0.32]*, p* = 0.007). Significant differences between the five time points were also found within each haplotype group: STAI-SKD scores increased from T1 to T2 in non-carriers (β = –2.24, 95% CI [–3.67, –0.81], *p* = 0.0001) and one-copy carriers (β = –1.01, 95% CI [–1.96, –0.05], *p* = 0.031), and both non-carriers and one-copy carriers showed a decrease in STAI-SKD scores from T2 to T3 (β = 2.17, 95% CI [0.71, 3.62], *p* = 0.0003 and β = 1.16, *95%* CI [0.18, 2.13], *p* = 0.008, respectively), T2 to T4 (β = 3.01, 95% CI [1.56, 4.47], *p* = 0.0001 and β = 1.66, 95% CI [0.71, 2.62], *p* = 0.0001, respectively), and T2 to T5 (β = 2.46, 95% CI [1.03, 3.89], *p* = 0.0001 and β = 1.89, 95% CI:[0.94, 2.84], *p* = 0.0001, respectively). A decrease in STAI-SKD scores was also found from T1 to T5 in one-copy carriers (β = 0.89, 95% CI [0.008, 1.76], *p* = 0.046), and from T3 to T4 (β = 1.52, 95% CI [0.29, 2.74]*, p* = 0.005) and T3 to T5 (β = 1.71, 95% CI [0.47, 2.94], *p* = 0.001) in two-copy carriers.

### Perinatal mood, oestrogen receptor genes and oestradiol

None of the interaction models showed significant results for the LRTs (see Table S7-8); therefore, no F-tests for fixed effects were carried out.

## Discussion

The present study investigated the interaction between ER gene variations, E2 levels and mood during the transition from pregnancy to postpartum using a longitudinal study design. The haplotypes CG and TA in *ESR1* were associated with perinatal mood disturbances. Women carrying two copies of the haplotype CG exhibited elevated depressive symptoms at 40 weeks of gestation and within 48 h after delivery, and elevated anxiety throughout the perinatal period, compared to those carrying one or no copies. Across all genetic subgroups, women with no copy of the haplotype TA showed the highest anxiety scores at 40 weeks of gestation and 8-12 weeks postpartum. No associations were found for the interaction between ER gene variations, E2 levels and perinatal mood disturbances.

Previous research has indicated that various genetic variations are associated with the onset of perinatal depression, specifically in the last months of pregnancy and up to eight weeks postpartum (Figueiredo et al. 2015). In line with this, we found an interaction between haplotypes, time and perinatal mood disturbances, indicating that these haplotypes affect mood symptoms, particularly around 40 weeks of gestation and in the initial 48 h following delivery, and between 8–12 weeks postpartum. Thus, these variations may serve as potential biomarkers for mood disorders during specific perinatal time points. However, exploratory follow-up analyses did not reveal any association with the potential diagnosis of perinatal depression at any time point. Importantly, these findings should be interpreted with caution given the rather healthy sample and thus the low proportion of women meeting the cut-off for a potential diagnosis of perinatal depression, also in comparison to similar studies (e.g., Pinsonneault et al. 2013). Therefore, future studies with larger, well-powered groups of women diagnosed with perinatal depression are needed to validate these associations and further explore their potential clinical relevance. While genetic susceptibility to perinatal mood disorders remains constant throughout life, its effects may become more pronounced in response to environmental factors and biological changes that occur during the perinatal period (Guintivano et al. 2018). Considering the unique course of E2 levels across the peripartum, we hypothesised that ER gene variations would have distinct implications for mood symptoms depending on the E2 levels at different time points. However, no associations emerged between ER gene variations, E2 levels and perinatal mood disturbances. One explanation for this might lie in the fact that we employed absolute E2 levels rather than fluctuation measures. A recent study by our workgroup provided initial evidence of an interplay between ER genes and E2 fluctuations (Grub et al. 2024). In detail, ER gene variations modulated the effect of E2 fluctuations on menopausal symptom trajectories, including psychological, somatic-vegetative and urogenital symptoms.

So far, genetic variations encoding for ER-α have been more frequently associated with mood symptoms in women compared to variations encoding for ER-β, suggesting that ER-α plays a more important role in emotional processes than other ERs (Ryan and Ancelin 2012; Li et al. 2022). Similarly, we identified an association between perinatal mood disturbances and variations encoding for ER-α, but not for ER-β and GPER. Although ER-α and ER-β are similar in structure and function, several differences may modulate their effects on mood symptoms (Ascenzi et al. 2006; Sundermann et al. 2010). One such difference is their distinct neuronal expression pattern: ER-α is more dominantly expressed in the amygdala and hypothalamus than ER-β, which may explain its more important role in mood regulation (Osterlund and Hurd 2001; Ostlund et al. 2003). In contrast, GPERs are expressed in the hippocampus, cortex and hypothalamus and exhibit a structure that is more distinct from both ER-α and ER-β (Prossnitz and Barton 2011; Prossnitz and Hathaway 2015). Several factors may explain the lack of association between GPER and perinatal mood disturbances. One reason may lie in the use of the EPDS to assess subjective mood, as this scale aggregates depressive symptoms over a period of two weeks. Given that GPERs primarily mediate oestrogen’s rapid non-genomic effects (Fuentes and Silveyra 2019), the EPDS scores may not accurately reflect the rapid response to oestrogen mediated by GPERs. However, we did not find an association between *GPER* and anxiety, which was assessed using a current-state anxiety questionnaire. Another potential reason might be the limited number of investigated SNPs in *GPER*. Accordingly, other genetic variations in *GPER*, which were not investigated in the present study, may be involved in perinatal mood disturbances.

A recent meta-analysis revealed that the C allele of rs2234693 and the G allele of rs9340799 are associated with an increased risk of depression in women (Li et al. 2022). In line with this, we found that two-copy carriers of the haplotype CG, corresponding to the genotype CC of rs2234693 and GG of rs9340799, appeared to be susceptible to perinatal mood disturbances. In contrast, carriers of one or more copies of the haplotype TA, which refers to at least one T allele of rs2234693 and one A allele of rs9340799, seemed to be less susceptible to perinatal mood disturbances. Interestingly, both SNPs are located in a non-coding region of the gene. Consequently, rather than directly encoding the amino acid sequence, they modify the transcription of *ESR1* through altered transcription factor binding (Maney 2017). For instance, Herrington et al. (2002) demonstrated that the C allele of rs2234693 created a functional binding site for the transcription factor B-Myb, thereby amplifying *ESR1* transcription. Therefore, it can be hypothesised that the upregulation of ER-α expression through the C allele of rs2234693 may increase sensitivity to oestrogen and thus susceptibility to perinatal mood disturbances. Findings by Mehta et al. (2014) also point to an increased sensitivity to oestrogen in women with PPD. Notably, the authors found enrichment of transcription factor binding sites for ER-α in affected women compared to healthy controls, despite no differences in oestrogen levels. However, the specific functionality and implicated biological pathways of these variations remain inconclusive (Sundermann et al. 2010; Maney 2017). Thus, it can be argued that these variations may not directly affect oestrogen signalling. Moreover, the above-mentioned associations may arise not from the variations investigated but rather from their linkage with another functional variation that is associated with perinatal mood disturbances. For instance, both rs2234693 and rs9340799 were found to be in LD with the SNP rs2077647 in *ESR1*, which in turn was associated with both depressive symptoms and PPD within the first 12 weeks postpartum (Pinsonneault et al. 2013; Tan et al. 2018). However, these associations did not remain significant after correction for multiple testing, suggesting that the initial results may have been affected by false positives.

Nevertheless, several limitations of this study need to be considered. First, our study sample consisted mainly of healthy participants with low mood symptom scores, which restricts the ability to generalise our findings to individuals with clinically relevant mood disorders. For instance, two-copy carriers of the haplotype CG exhibited the highest EPDS scores, with a mean of around 9 points shortly after birth, which remains below the proposed cut-off score of 12/13 for depression in the original validation study (Cox et al. 1987) and below the proposed cut-off score of 11 to maximise combined sensitivity and specificity for depression (Levis et al. 2020). In addition, although exploratory follow-up analyses were conducted to examine the association with perinatal depression, the ability to detect significant associations may have been limited by the small number of women meeting the cut-off (EPDS ≥ 11) for a potential clinically relevant diagnosis of perinatal depression. Second, we addressed population stratification only by including women of self-reported European ancestry rather than by genotyping ancestry information markers, which may have introduced biases or confounding due to inaccuracies in self-reported data. Third, the interaction analysis was conducted with a smaller sample size than the genetic analysis, thereby reducing the statistical power. Although imputation was applied to address the missing E2 data, the high percentage of missing values (32.8%), of which 48.8% were above the sensitivity threshold, may have introduced bias (Kleinke 2018). Notably, our data showed comparable to higher rates of missing values than other studies conducting in-home saliva assessments during the perinatal period (e.g., Iliadis et al. 2015; Murphy et al. 2022). This can be attributed to at least two reasons. First, non-compliance with in-home saliva collection, which is both common and highly variable during pregnancy, may have been exacerbated by the large number of required samples (Kudielka et al. 2007; Moeller et al. 2014). Second, the sensitivity threshold of the ELISA (>64 pg/mL) overlapped with the salivary E2 range during the third trimester (24–75 pg/mL), which increased the likelihood of samples not being detected, particularly during the first two assessment time points (Dukic and Ehlert 2023).

In conclusion, the present study indicates that *ESR1* variations, which may increase ER-α’s sensitivity to oestrogen, are associated with an elevated susceptibility to perinatal mood disturbances. Moreover, the findings suggest that this effect is dependent on time, and thus potentially on different E2 levels. However, the inclusion of an interaction with E2 levels did not explain perinatal mood disturbances. Therefore, sensitivity differences in ER-α seem to play a more important role in emotional processes than sensitivity differences in ER-β and GPER, independently of E2 levels, which might be explained by the receptor’s more dominant expression in the hypothalamus and amygdala. However, due to the correlational study design, the underlying mechanisms by which these variations may ultimately affect perinatal mood disturbances remain unclear. Nevertheless, our findings highlight the importance of inter- and intraindividual investigation of perinatal mood symptoms related to oestrogen sensitivity, which future studies should consider. Although evidence is still insufficient to establish these genetic variations as biomarkers for perinatal mood disorders, they provide a promising target for future research. However, more comprehensive studies are needed that consider not only individual genetic variations, but also combinations with additional variations, as well as interactions with further biopsychosocial factors. Such approaches may improve our understanding of perinatal mood disorders and ultimately contribute to improved early detection.

## Supplementary Material

Supplementary materials.docx

## Data Availability

The data that support the findings of this study are available from the corresponding author, U.E., upon reasonable request.

## References

[CIT0001] Andela M, Dewani D. 2023. Postpartum mood disorders: a short review. J South Asian Fed Obstet Gynaecol. 15(1):102–107. doi: 10.5005/jp-journals-10006-2170.

[CIT0002] Ascenzi P, Bocedi A, Marino M. 2006. Structure-function relationship of estrogen receptor alpha and beta: impact on human health. Mol Aspects Med. 27(4):299–402. doi: 10.1016/j.mam.2006.07.001. 16914190

[CIT0003] Barth C, Villringer A, Sacher J. 2015. Sex hormones affect neurotransmitters and shape the adult female brain during hormonal transition periods. Front Neurosci. 9:37. doi: 10.3389/fnins.2015.00037. 25750611 PMC4335177

[CIT0004] Bergant AM, Nguyen T, Heim K, Ulmer H, Dapunt O. 1998. Deutschsprachige Fassung und Validierung der “Edinburgh postnatal depression scale” [German language version and validation of the Edinburgh postnatal depression scale]. Dtsch Med Wochenschr. 123(3):35–40. doi: 10.1055/s-2007-1023895. 9472218

[CIT0005] Bloch M, Daly RC, Rubinow DR[R]. 2003. Endocrine factors in the etiology of postpartum depression. Compr Psychiatry. 44(3):234–246. doi: 10.1016/S0010-440X(03)00034-8. 12764712

[CIT0006] Champagne FA, Curley JP. 2008. Maternal regulation of estrogen receptor alpha methylation. Curr Opin Pharmacol. 8(6):735–739. doi: 10.1016/j.coph.2008.06.018. 18644464 PMC2612119

[CIT0007] Clark AG. 2004. The role of haplotypes in candidate gene studies. Genet Epidemiol. 27(4):321–333. doi: 10.1002/gepi.20025. 15368617

[CIT0008] Costas J, Gratacòs M, Escaramís G, Martín-Santos R, Diego Y, Baca-García E, Canellas F, Estivill X, Guillamat R, Guitart M, et al. 2010. Association study of 44 candidate genes with depressive and anxiety symptoms in post-partum women. J Psychiatr Res. 44(11):717–724. doi: 10.1016/j.jpsychires.2009.12.012. 20092830

[CIT0009] Cox JL, Holden JM, Sagovsky R. 1987. Detection of postnatal depression. Development of the 10-item Edinburgh Postnatal Depression Scale. Br J Psychiatry. 150(6):782–786. doi: 10.1192/bjp.150.6.782. 3651732

[CIT0010] Del Río JP, Alliende MI, Molina N, Serrano FG, Molina S, Vigil P. 2018. Steroid hormones and their action in women’s brains: the importance of hormonal balance. Front Public Health. 6:141. doi: 10.3389/fpubh.2018.00141. 29876339 PMC5974145

[CIT0011] Dennis C‑L, Falah-Hassani K, Shiri R. 2017. Prevalence of antenatal and postnatal anxiety: systematic review and meta-analysis. Br J Psychiatry. 210(5):315–323. doi: 10.1192/bjp.bp.116.187179. 28302701

[CIT0012] Dukic J, Ehlert U. 2023. Longitudinal course of sex steroids from pregnancy to postpartum. Endocrinology. 164(8):bqad108. doi: 10.1210/endocr/bqad108. 37450580 PMC10499333

[CIT0013] Dukic J, Johann A, Henninger M, Ehlert U. 2024. Estradiol and progesterone from pregnancy to postpartum: a longitudinal latent class analysis. Front Glob Womens Health. 5:1428494. doi: 10.3389/fgwh.2024.1428494. 39444825 PMC11496150

[CIT0014] El-Ibiary SY, Hamilton SP, Abel R, Erdman CA, Robertson PA, Finley PR. 2013. A pilot study evaluating genetic and environmental factors for postpartum depression. Innov Clin Neurosci. 10(9-10):15–22. PMC3849876 24307977

[CIT0015] Englert C, Bertrams A, Dickhäuser O. 2011. Entwicklung der Fünf-Item-Kurzskala STAI-SKD zur Messung von Zustandsangst. Zeitschrift Für Gesundheitspsychologie. 19(4):173–180. doi: 10.1026/0943-8149/a000049.

[CIT0016] Figtree GA, Noonan JE, Bhindi R, Collins P. 2009. Estrogen receptor polymorphisms: significance to human physiology, disease and therapy. Recent Pat DNA Gene Seq. 3(3):164–171. doi: 10.2174/187221509789318397. 19673701

[CIT0017] Figueiredo FP, Parada AP, Araujo LF, de Silva WA, Del-Ben CM. 2015. The influence of genetic factors on peripartum depression: a systematic review. J Affect Disord. 172:265–273. doi: 10.1016/j.jad.2014.10.016. 25451426

[CIT0018] Fuentes N, Silveyra P. 2019. Estrogen receptor signaling mechanisms. Adv Protein Chem Struct Biol. 116:135–170. doi: 10.1016/bs.apcsb.2019.01.001. 31036290 PMC6533072

[CIT0019] Furtado M, Chow CHT, Owais S, Frey BN, van Lieshout RJ. 2018. Risk factors of new onset anxiety and anxiety exacerbation in the perinatal period: a systematic review and meta-analysis. J Affect Disord. 238:626–635. doi: 10.1016/j.jad.2018.05.073. 29957480

[CIT0020] Grigoriadis S, Graves L, Peer M, Mamisashvili L, Tomlinson G, Vigod SN, Dennis C, Steiner L, Brown M, Cheung C, et al. 2018. Maternal anxiety during pregnancy and the association with adverse perinatal outcomes: systematic review and meta-analysis. J Clin Psychiatry. 79(5):17r12011. doi: 10.4088/JCP.17r12011. 30192449

[CIT0021] Grub J, Willi J, Süss H, Ehlert U. 2024. The role of estrogen receptor gene polymorphisms in menopausal symptoms and estradiol levels in perimenopausal women—findings from the Swiss Perimenopause Study. Maturitas. 183:107942. doi: 10.1016/j.maturitas.2024.107942. 38412592

[CIT0022] Guintivano J, Manuck T, Meltzer-Brody S. 2018. Predictors of postpartum depression: a comprehensive review of the last decade of evidence. Clin Obstet Gynecol. 61(3):591–603. doi: 10.1097/GRF.0000000000000368. 29596076 PMC6059965

[CIT0023] Hazell GGJ, Yao ST, Roper JA, Prossnitz ER, O’Carroll AM, Lolait SJ. 2009. Localisation of GPR30, a novel G protein-coupled oestrogen receptor, suggests multiple functions in rodent brain and peripheral tissues. J Endocrinol. 202(2):223–236. doi: 10.1677/JOE-09-0066. 19420011 PMC2710976

[CIT0024] Herrington DM, Howard TD, Brosnihan KB, McDonnell DP, Li X, Hawkins GA, Reboussin DM, Xu J, Zheng SL, Meyers DA, et al. 2002. Common estrogen receptor polymorphism augments effects of hormone replacement therapy on E-selectin but not C-reactive protein. Circulation. 105(16):1879–1882. doi: 10.1161/01.CIR.0000016173.98826.88. 11997270

[CIT0025] Hua H, Zhang H, Kong Q, Jiang Y. 2018. Mechanisms for estrogen receptor expression in human cancer. Exp Hematol Oncol. 7(1):24. doi: 10.1186/s40164-018-0116-7. 30250760 PMC6148803

[CIT0026] Hwang WJ, Lee TY, Kim NS, Kwon JS. 2020. The role of estrogen receptors and their signaling across psychiatric disorders. Int J Mol Sci. 22(1):373. doi: 10.3390/ijms22010373. 33396472 PMC7794990

[CIT0027] Iliadis SI, Comasco E, Sylvén S, Hellgren C, Sundström Poromaa I, Skalkidou A. 2015. Prenatal and postpartum evening salivary cortisol levels in association with peripartum depressive symptoms. PLoS One. 10(8):e0135471. doi: 10.1371/journal.pone.0135471. 26322643 PMC4556108

[CIT0028] Johann A, Dukic J, Rothacher Y, Ehlert U. 2023. Trajectories of reproductive transition phase mood disorder from pregnancy to postpartum: a Swiss longitudinal study. Womens Health (Lond). 19:17455057221147391. doi: 10.1177/17455057221147391. 36748405 PMC9909046

[CIT0029] Johann A, Ehlert U. 2020. The study protocol: neuroendocrinology and (epi-) genetics of female reproductive transition phase mood disorder—an observational, longitudinal study from pregnancy to postpartum. BMC Pregnancy Childbirth. 20(1):609. doi: 10.1186/s12884-020-03280-5. 33036563 PMC7545379

[CIT0030] Kleinke K. 2018. Multiple imputation by predictive mean matching when sample size is small. Methodology. 14(1):3–15. doi: 10.1027/1614-2241/a000141.

[CIT0031] Krolick KN, Zhu Q, Shi H. 2018. Effects of estrogens on central nervous system neurotransmission: implications for sex differences in mental disorders. Prog Mol Biol Transl Sci. 160:105–171. doi: 10.1016/bs.pmbts.2018.07.008. 30470289 PMC6737530

[CIT0032] Kudielka BM, Hawkley LC, Adam EK, Cacioppo JT. 2007. Compliance with ambulatory saliva sampling in the Chicago health, aging, and social relations study and associations with social support. Ann Behav Med. 34(2):209–216. doi: 10.1007/BF02872675. 17927559

[CIT0033] Kuijper EAM, Ket JCF, Caanen MR, Lambalk CB. 2013. Reproductive hormone concentrations in pregnancy and neonates: a systematic review. Reprod Biomed Online. 27(1):33–63. doi: 10.1016/j.rbmo.2013.03.009. 23669015

[CIT0034] Letourneau NL, Dennis CL, Cosic N, Linder J. 2017. The effect of perinatal depression treatment for mothers on parenting and child development: a systematic review. Depress Anxiety. 34(10):928–966. doi: 10.1002/da.22687. 28962068

[CIT0035] Levis B, Negeri Z, Sun Y, Benedetti A, Thombs BD, DEPRESsion Screening Data (DEPRESSD) EPDS Group 2020. Accuracy of the Edinburgh Postnatal Depression Scale (EPDS) for screening to detect major depression among pregnant and postpartum women: systematic review and meta-analysis of individual participant data. BMJ. 371:m4022. doi: 10.1136/bmj.m4022. 33177069 PMC7656313

[CIT0036] Li C, Xie M, Wang W, Liu Y, Liao D, Yin J, Huang H. 2022. Association between polymorphisms in estrogen receptor genes and depression in women: a meta-analysis. Front Genet. 13:936296. doi: 10.3389/fgene.2022.936296. 35928452 PMC9343944

[CIT0037] Maney DL. 2017. Polymorphisms in sex steroid receptors: from gene sequence to behavior. Front Neuroendocrinol. 47:47–65. doi: 10.1016/j.yfrne.2017.07.003. 28705582 PMC6312198

[CIT0038] Mehta D, Newport J, Frishman G, Kraus L, Rex-Haffner M, Ritchie JC, Lori A, Knight BT, Stagnaro E, Ruepp A, et al. 2014. Early predictive biomarkers for postpartum depression point to a role for estrogen receptor signaling. Psychol Med. 44(11):2309–2322. doi: 10.1017/s0033291713003231. 24495551

[CIT0039] Mehta D, Rex-Haffner M, Søndergaard HB, Pinborg A, Binder EB, Frokjaer VG. 2019. Evidence for oestrogen sensitivity in perinatal depression: pharmacological sex hormone manipulation study. Br J Psychiatry. 215(3):519–527. doi: 10.1192/bjp.2018.234. 30457060

[CIT0040] Moeller J, Lieb R, Meyer AH, Loetscher KQ, Krastel B, Meinlschmidt G. 2014. Improving ambulatory saliva-sampling compliance in pregnant women: a randomized controlled study. PLoS One. 9(1):e86204. doi: 10.1371/journal.pone.0086204. 24465958 PMC3899170

[CIT0041] Murphy HR, Gu Y, Wu Q, Brunner J, Panisch LS, Best M, Arnold MS, Duberstein ZT, Putzig J, Carnahan J, et al. 2022. Prenatal diurnal cortisol: normative patterns and associations with affective symptoms and stress. Psychoneuroendocrinology. 143:105856. doi: 10.1016/j.psyneuen.2022.105856. 35797838

[CIT0042] Orsolini L, Valchera A, Vecchiotti R, Tomasetti C, Iasevoli F, Fornaro M, Berardis D, de Perna G, Pompili M, Bellantuono C, et al. 2016. Suicide during perinatal period: epidemiology, risk factors, and clinical correlates. Front Psychiatry. 7:138. doi: 10.3389/fpsyt.2016.00138. 27570512 PMC4981602

[CIT0043] Osterlund MK, Hurd YL. 2001. Estrogen receptors in the human forebrain and the relation to neuropsychiatric disorders. Prog Neurobiol. 64(3):251–267. doi: 10.1016/S0301-0082(00)00059-9. 11240308

[CIT0044] Ostlund H, Keller E, Hurd YL. 2003. Estrogen receptor gene expression in relation to neuropsychiatric disorders. Ann N Y Acad Sci. 1007(1):54–63. doi: 10.1196/annals.1286.006. 14993040

[CIT0045] Payne JL, Palmer JT, Joffe H. 2009. A reproductive subtype of depression: conceptualizing models and moving toward etiology. Harv Rev Psychiatry. 17(2):72–86. doi: 10.1080/10673220902899706. 19373617 PMC3741092

[CIT0046] Pinsonneault JK, Sullivan D, Sadee W, Soares CN, CN, Hampson E, Steiner M. 2013. Association study of the estrogen receptor gene ESR1 with postpartum depression—a pilot study. Arch Womens Ment Health. 16(6):499–509. doi: 10.1007/s00737-013-0373-8. 23917948 PMC3833886

[CIT0047] Prossnitz ER, Barton M. 2011. The G-protein-coupled estrogen receptor GPER in health and disease. Nat Rev Endocrinol. 7(12):715–726. doi: 10.1038/nrendo.2011.122. 21844907 PMC3474542

[CIT0048] Prossnitz ER, Hathaway HJ. 2015. What have we learned about GPER function in physiology and disease from knockout mice? J Steroid Biochem Mol Biol. 153:114–126. doi: 10.1016/j.jsbmb.2015.06.014. 26189910 PMC4568147

[CIT0049] R Core Team. R: a language and environment for statistical computing. https://www.R-project.org/

[CIT0050] Ryan J, Ancelin M-L. 2012. Polymorphisms of estrogen receptors and risk of depression: therapeutic implications. Drugs. 72(13):1725–1738. doi: 10.2165/11635960-000000000-00000. 22901010

[CIT0051] Schaid DJ, Rowland CM, Tines DE, Jacobson RM, Poland GA. 2002. Score tests for association between traits and haplotypes when linkage phase is ambiguous. Am J Hum Genet. 70(2):425–434. doi: 10.1086/338688. 11791212 PMC384917

[CIT0052] Schiller CE, Walsh CE, Eisenlohr-Moul E, Prim TA, Dichter J, Schiff GS, Bizzell L, Slightom J, Richardson SL, Belger EC, et al. 2022. Effects of gonadal steroids on reward circuitry function and anhedonia in women with a history of postpartum depression. J Affect Disord. 314:176–184. doi: 10.1016/j.jad.2022.06.078. 35777494 PMC9605402

[CIT0053] Schiller CE, Meltzer-Brody S, Rubinow DR. 2015. The role of reproductive hormones in postpartum depression. CNS Spectr. 20(1):48–59. doi: 10.1017/S1092852914000480. 25263255 PMC4363269

[CIT0054] Slatkin M. 2008. Linkage disequilibrium–understanding the evolutionary past and mapping the medical future. Nat Rev Genet. 9(6):477–485. doi: 10.1038/nrg2361. 18427557 PMC5124487

[CIT0055] Slomian J, Honvo G, Emonts P, Reginster JY, Bruyère O. 2019. Consequences of maternal postpartum depression: a systematic review of maternal and infant outcomes. Womens Health (Lond). 15:1745506519844044. doi: 10.1177/1745506519844044. 31035856 PMC6492376

[CIT0056] Soares CN, Zitek B. 2008. Reproductive hormone sensitivity and risk for depression across the female life cycle: a continuum of vulnerability? J Psychiatry Neurosci. 33(4):331–343. 18592034 PMC2440795

[CIT0057] Sundermann EE, Maki PM, Bishop JR. 2010. A review of estrogen receptor alpha gene (ESR1) polymorphisms, mood, and cognition. Menopause. 17(4):874–886. doi: 10.1097/gme.0b013e3181df4a19. 20616674 PMC2901885

[CIT0058] Tan EC, Lim HW, Chua TE, Tan HS, Lee TM, Chen HY. 2018. Investigation of variants in estrogen receptor genes and perinatal depression. Neuropsychiatr Dis Treat. 14:919–925. doi: 10.2147/NDT.S160424. 29636617 PMC5880413

[CIT0059] Vannuccini S, Bocchi C, Severi FM, Challis JR, Petraglia F. 2016. Endocrinology of human parturition. Ann Endocrinol (Paris). 77(2):105–113. doi: 10.1016/j.ando.2016.04.025. 27155774

[CIT0060] Wang Z, Liu J, Shuai H, Cai Z, Fu X, Liu Y, Yang Xiao X, Zhang W, Krabbendam E, Liu S, et al. 2021. Mapping global prevalence of depression among postpartum women. Transl Psychiatry. 11(1):543. doi: 10.1038/s41398-021-01663-6. 34671011 PMC8528847

[CIT0061] Yim IS, Tanner Stapleton LR, Guardino CM, Hahn-Holbrook J, Dunkel Schetter C. 2015. Biological and psychosocial predictors of postpartum depression: systematic review and call for integration. Annu Rev Clin Psychol. 11(1):99–137. doi: 10.1146/annurev-clinpsy-101414-020426. 25822344 PMC5659274

[CIT0062] Yu Y, Liang H-F, Chen J, Li Z-B, Han Y-S, Chen J-X, Li J-C. 2021. Postpartum depression: current status and possible identification using biomarkers. Front Psychiatry. 12:620371. doi: 10.3389/fpsyt.2021.620371. 34211407 PMC8240635

[CIT0063] Zhao XH, Zhang ZH. 2020. Risk factors for postpartum depression: an evidence-based systematic review of systematic reviews and meta-analyses. Asian J Psychiatr. 53:102353. doi: 10.1016/j.ajp.2020.102353. 32927309

