# Supplementary material for: The complex interaction between oestrogen receptor genes, oestradiol, and perinatal mood
Source: Dialogues Clin Neurosci. 2025 Mar 21;27(1):24–33. doi: 10.1080/19585969.2025.2482126 (PMC11934172; doi:10.1080/19585969.2025.2482126)
Supplement: Supplementary materials.docx [file TDCN_A_2482126_SM7722.docx]

**Supplementary materials**

**Figure S1……………………………………………………………………………………......………2**

**Table S1…………..……………………………………………………………………………..………3**

**Table S2…………..……………………………………………………………………………..………4**

**Table S3………….……………………………………………………………………………..….……5**

**Table S4…………..……………………………………………………………………………..………6**

**Table S5…………..……………………………………………………………………………..………7**

**Table S6…………..……………………………………………………………………………..………8**

**Table S7…………..……………………………………………………………………………..………9**

**Table S8…………..……………………………………………………………………………………10**

**Figure S1**

Flow chart of participant selection for the study.


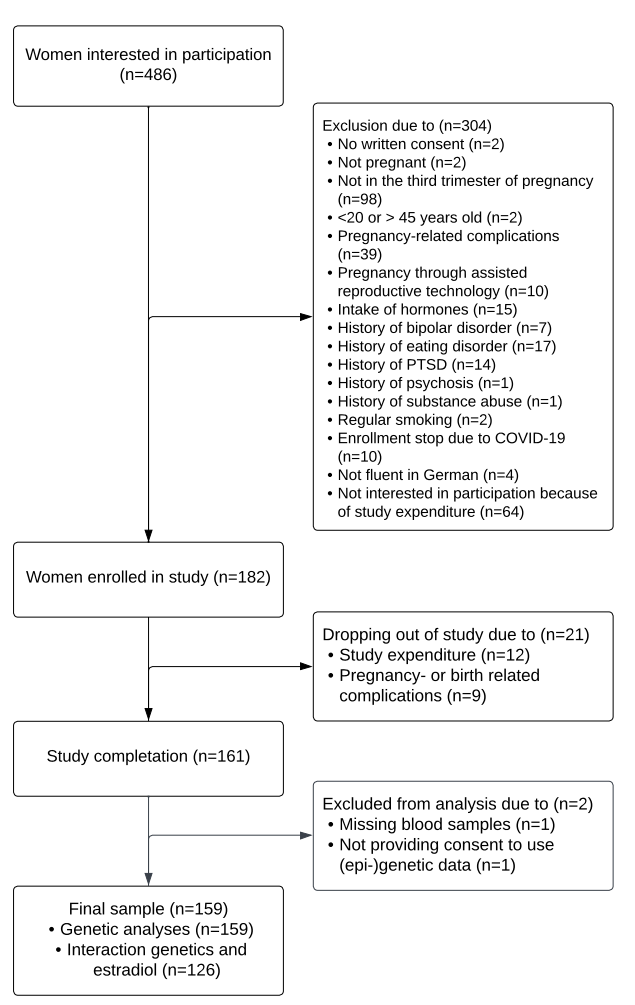


| **Table S1**  Sociodemographic and health-related sample characteristics (n=159). | |
| --- | --- |
| Variable | N (%) or M (SD)^a^ |
| Age | 32.7 (4.0) |
| Nationality  Swiss  German  Other | 114 (71.7)  22 (13.8)  23 (14.5) |
| Education  Vocational school  Higher vocational school  General University entrance qualification  University  Other | 20 (12.5)  6 (3.8)  6 (3.8)  110 (69.2)  17 (10.7) |
| Relationship status  Single  In a relationship, but not cohabitating  In a relationship and cohabitating  Married and cohabitating  Married, not cohabitating (separation) | 1 (0.6)  2 (1.3)  63 (39.6)  92 (57.9)  1 (0.6) |
| History of depression  Yes  No  Unknown | 43 (27.1)  101 (63.5)  15 (9.4) |
| Psychiatric disorder in family  Yes  No  Unknown | 55 (34.6)  74 (46.5)  30 (18.9) |
| PPD of a female family member  Yes  No | 20 (12.6)  139 (87.4) |
| Parity  Nulliparous  One child  Two children  Three or more children | 88 (55.4)  42 (26.4)  13 (8.2)  16 (10.0) |
| Pregnancy planned  Yes  No | 127 (79.9)  32 (20.1) |
| Delivery mode  Vaginal  Cesarean section  Unknown | 112 (70.5)  42 (26.4)  5 (3.1) |
| Breastfeeding  Yes  No  Unknown | 117 (73.6)  37 (23.3)  5 (3.1) |
| Note. ^a^Mean and standard deviation (SD) are shown for continuous characteristics, and frequency and percentage (%) for categorical characteristics. | |

| **Table S2**  Mean mood scores and estradiol levels with standard deviations for each assessment time point. | | | | |  |  |
| --- | --- | --- | --- | --- | --- | --- |
| Variable | T1 | T2 | T3 | T4 | | T5 |
|  | M (SD) | M (SD) | M (SD) | M (SD) | | M (SD) |
| EPDS | 4.8 (4.3) | 4.4 (4.4) | 5.5 (5.1) | 5.2 (4.5) | | 4.0 (4.4) |
| STAI-SKD | 7.7 (2.4) | 8.7 (2.5) | 7.7 (2.7) | 6.9 (2.4) | | 6.9 (2.6) |
| Estradiol (pg/mL) | 43.1 (7.8) | 41.7 (7.9) | 13.1 (7.9) | 3.9 (2.9) | | 3.7 (1.9) |
| Note. N=159. EPDS, Edinburgh Postnatal Depression Scale, SD, standard deviation, STAI-SKD, short form of the state subscale of the State-Trait Anxiety Inventory, T1, 34-36 weeks of gestation, T2, 40 weeks of gestation, T3, within the first 48 hours after delivery, T4, 4-8 weeks postpartum, T5, 8-12 weeks postpartum. | | | | | | |

| **Table S3**  Absolute frequencies and percentages of genotype and allele distributions for each single polymorphism. | | | | | | | | |  |
| --- | --- | --- | --- | --- | --- | --- | --- | --- | --- |
| SNP | Gene | Variation | Genotype |  |  | Allele |  | X^2^ | HWE P-value |
|  |  |  | Homozygous common | Heterozygous | Homozygous  rare | Common | Minor |  |  |
| rs2234693 | ESR1 | T>C | 42 (26%) | 82 (52%) | 35 (22%) | 166 (52%) | 152 (48%) | 0.17 | 0.91 |
| rs9340799 | ESR1 | A>G | 62 (39%) | 76 (48%) | 21 (13%) | 200 (63%) | 118 (37%) | 0.09 | 0.95 |
| rs1256049 | ESR2 | C>T | 142 (89%) | 16 (10%) | 1 (0.6%) | 300 (94%) | 18 (6%) | 0.53 | 0.76 |
| rs4986938 | ESR2 | C>T | 73 (46%) | 66 (42%) | 20 (13%) | 212 (67%) | 106 (33%) | 0.69 | 0.70 |
| rs3808350 | GPER | A>G | 70 (44%) | 68 (43%) | 20 (13%) | 208 (66%) | 108 (34%) | 0.29 | 0.86 |
| Note. HWE, Hardy-Weinberg equilibrium, SNP, single nucleotide polymorphism. | | | | | | | | | |

| **Table S4**  Absolute frequencies and percentages (in brackets) of the reconstructed haplotypes for ESR1 and ESR2. | | | | | |
| --- | --- | --- | --- | --- | --- |
| Gene | SNPs | Haplotype | Haplotype frequency | | |
|  |  |  | 0 | 1 | 2 |
| ESR1 | rs2234693, rs9340799 | CG  TA  CA | 62 (39.0%)  35 (22.0%)  128 (80.5%) | 76 (47.8%)  82 (51.6%)  28 (17.6%) | 21 (13.2%)  42 (26.4%)  3 (1.9%) |
| ESR2 | rs1256049, rs4986938 | CC  CT  TC  TT | 25 (15.7%)  73 (45.9%)  142 (89.3%)  158 (99.3%) | 74 (46.5%)  66 (41.5%)  16 (10.1%)  1 (0.7%) | 60 (37.8%)  20 (12.6%)  1 (0.6%)  0 (0%) |
| Note. SNP, single nucleotide polymorphism. | | | | | |

| **Table S5**  Likelihood ratio test results of the comparison between the genetic and basic model. | | | |
| --- | --- | --- | --- |
| Gene | Haplotype/SNP | EPDS | STAI-SKD |
|  |  | *p*-value | *p*-value |
| ESR1 | CG  TA  CA | 0.001754^a^  0.01757  0.2441 | 0.0001506^a^  0.0009971^a^  0.2243 |
| ESR2 | CC  CT  TC | 0.06394  0.1721  0.07903 | 0.1944  0.48  0.2939 |
| GPER | rs3808350 | 0.6422 | 0.9423 |
| Note. EPDS, Edinburgh Postnatal Depression Scale, STAI-SKD, short form of the state subscale of the State-Trait Anxiety Inventory.  ^a^ significant after Bonferroni correction (*p*=0.00178). | | | |

| **Table S6**  Likelihood ratio test results of the comparison between the genetic and basic model with age and depression history as covariates. | | | |
| --- | --- | --- | --- |
| Gene | Haplotype/SNP | EPDS | STAI-SKD |
|  |  | *p*-value | *p*-value |
| ESR1 | CG  TA  CA | 0.002176  0.01286  0.3515 | 0.0002735^a^  0.001055^a^  0.2339 |
| ESR2 | CC  CT  TC | 0.08006  0.194  0.09127 | 0.2005  0.4911  0.3251 |
| GPER | rs3808350 | 0.5975 | 0.8941 |
| Note. EPDS, Edinburgh Postnatal Depression Scale, STAI-SKD, short form of the state subscale of the State-Trait Anxiety Inventory. ^a^ significant after Bonferroni correction (*p*=0.00178). | | | |

| **Table S7**  Likelihood ratio test results of the comparison between the genetic and interaction model. | | | |
| --- | --- | --- | --- |
| Gene | Haplotype/SNP | EPDS | STAI-SKD |
|  |  | *p*-value | *p*-value |
| ESR1 | CG  TA  CA | 0.395  0.4819  0.1544 | 0.299  0.4716  0.4383 |
| ESR2 | CC  CT  TC | 0.3022  0.2914  0.1514 | 0. 8471  0.4578  0.3833 |
| GPER | rs3808350 | 0.118 | 0.2903 |
| Note. EPDS, Edinburgh Postnatal Depression Scale, STAI-SKD, short form of the state subscale of the State-Trait Anxiety Inventory. Significance level after Bonferroni correction *p* ≤ 0.00178. | | | |

| **Table S8**  Likelihood ratio test results of the comparison between the interaction and genetic model with age and depression history as covariates. | | | |
| --- | --- | --- | --- |
| Gene | Haplotype/SNP | EPDS | STAI-SKD |
|  |  | *p*-value | *p*-value |
| ESR1 | CG  TA  CA | 0.4021  0.4941  0.1484 | 0.3161  0.4927  0.4618 |
| ESR2 | CC  CT  TC | 0.2923  0.2921  0.1509 | 0.8532  0.4803  0.4058 |
| GPER | rs3808350 | 0.1119 | 0.3094 |
| Note. EPDS, Edinburgh Postnatal Depression Scale, STAI-SKD, short form of the state subscale of the State-Trait Anxiety Inventory.  Significance level after Bonferroni correction p ≤ 0.00178. | | | |

| **Table S9**  Positive screening results for perinatal depression across the five assessment time points. | | | | | | |
| --- | --- | --- | --- | --- | --- | --- |
| EPDS threshold | T1 (n=159) | T2 (n=115) |  | T3 (n=142) | T4 (n=152) | T5 (n=154) |
|  | N (%) | N (%) | | N (%) | N (%) | N (%) |
| ≥ 11 | 17 (12.1) | 12 (10.4) | | 25 (17.6) | 19 (12.5) | 15 (9.7) |
| Note. EPDS, Edinburgh Postnatal Depression Scale, T1, 34-36 weeks of gestation, T2, 40 weeks of gestation, T3, within the first 48 hours after delivery, T4, 4-8 weeks postpartum, T5, 8-12 weeks postpartum. | | | | | | |

| **Table S10**  Logistic regression results for perinatal depression diagnosis across the five time points. | | | | | | |
| --- | --- | --- | --- | --- | --- | --- |
| Haplotype | Frequency | T1 | T2 | T3 | T4 | T5 |
|  |  | *p*-value | *p*-value | *p*-value | *p*-value | *p*-value |
| CG | 0  1  2 | 0.985  0.873  0.138 | 0.142  0.915  0.192 | 0.238  0.439  0.053 | 0.909  0.117  0.857 | 0.344  0.559  0.851 |
| Note. EPDS, Edinburgh Postnatal Depression Scale, T1, 34-36 weeks of gestation, T2, 40 weeks of gestation, T3, within the first 48 hours after delivery, T4, 4-8 weeks postpartum, T5, 8-12 weeks postpartum. Perinatal depression cases were defined by the EPDS cut-off score of ≥ 11, and controls were defined by the EPDS cut-off score of < 11. All analyses were adjusted for age and history of depression. | | | | | | |
|  |  |  |  |  |  |  |
